# Tachyarrhythmia improved by management of low back pain in a patient with delayed diagnosis of infective spondylodiscitis: A case report

**DOI:** 10.1177/2050313X20952996

**Published:** 2020-08-23

**Authors:** Koshi Ota, Naoya Onishi, Kensuke Fujii, Eriko Nakamura, Yasuo Oishi, Masahiro Oka, Kanna Ota, Yohei Sano, Hiroki Yokoyama, Akira Takasu

**Affiliations:** 1 Department of Emergency Medicine, Osaka Medical College, Takatsuki, Japan; 2 Nonaka Clinic, Osaka, Japan

**Keywords:** Critical care/emergency medicine, infectious diseases, anesthesia/pain

## Abstract

A 77-year-old man presented to the emergency room with a 1-month history of persistent low back pain with the absence of vital sign abnormalities. On several previous orthopedic surgery clinic visits, pathological back pain had not been considered and pain killers had been prescribed because he had low back pain due to lumbar spinal canal stenosis. He was admitted to the intensive care unit for infectious spondylodiscitis and infective endocarditis with disseminated abscess caused by methicillin-resistant *Staphylococcus aureus*. Shock refractory tachyarrhythmia could not be managed with antiarrhythmic agent in the intensive care unit. Intractable low back pain and persistent tachyarrhythmia were adequately managed by pain control with fentanyl in the intensive care unit. Infectious spondylodiscitis and infective endocarditis were effectively managed with anti–methicillin-resistant *Staphylococcus aureus* drugs, initially in rotational usage, but the patient died of extended-spectrum beta-lactamase-producing *Escherichia coli* pneumonia on day 50 of hospitalization. Infectious spondylodiscitis should have been considered for persistent low back pain with hemodialysis, fever, and a history of device implantation. Pain management may be necessary for persistent tachycardia that proves unresponsive to usual antiarrhythmic medications.

## Introduction

Low back pain (LBP) is one of the most prevalent musculoskeletal disorders, affecting more than 90% of the population at some point in their lives.^1^ LBP is the most common musculoskeletal problem in males and the second-most common in females in Japan. LBP and its disability typically improve rapidly within weeks,^2^ but approximately 1% of patients who visit the emergency room (ER) have been reported to have serious diseases, including vertebral fracture, cancer, infection, cauda equina syndrome, and inflammatory arthritis.^3^ Infectious spondylodiscitis and vertebral osteomyelitis/discitis are particularly important as causes of LBP. Known risk factors for infectious spondylodiscitis include diabetes mellitus, alcoholism, hepatic cirrhosis, immunosuppression, intravenous drug use, malignancy, and renal failure being managed with hemodialysis (HD).^4^ HD has been identified as a risk factor for both infectious spondylodiscitis and infective endocarditis (IE), due to the repeated vascular punctures and contamination of the dialysis water purification system.^5,6^

Severe pain might be associated with tachycardia,^7^ if acute pain precipitates a stress response appearing physiologically as increases in heart rate, blood pressure, and plasma cortisol levels.^7^ One study of pediatric patients with abdominal pain revealed that tachycardia increased the relative risk of life-threatening diseases as much as 3.7-fold.^8^ Persistent tachyarrhythmia warrants immediate stabilization.

Here, we report the case of a patient with LBP due to infectious spondylodiscitis and IE who presented with intractable tachyarrhythmia that was well managed by pain control with fentanyl. The patient and his wife provided written, informed consent to publish the details of his condition. We also have obtained written informed consent from the legally authorized representatives (his wife) of the deceased subject for publication of this case report.

## Case presentation

A 77-year-old Japanese man had presented to the ER the previous day, with a 1-month history of persistent LBP and the absence of vital sign abnormalities. An orthopedic surgeon had examined him several times previously and had prescribed acetaminophen for pain management. The patient returned to the ER with worsened LBP around midnight the next day. He had a medical history of chronic kidney disease being managed with HD three times a week, hypertension, diabetes mellitus, paroxysmal atrial fibrillation, cervical spondylotic myelopathy, and lumbar spinal canal stenosis for which he had undergone posterior lumbar interbody fusion (PLIF) with lumbar interbody fusion device implantation.

**Figure 1.**
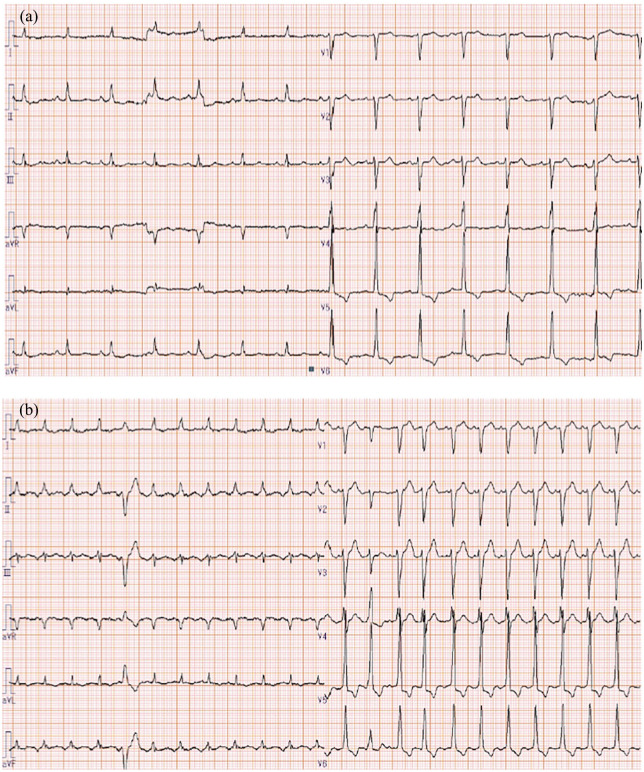
(a) Electrocardiogram on arrival in the emergency room (ER) shows a narrow-complex rhythm with ST-segment depression in leads II, V4–V6. Sinus P-waves are seen in leads II, III, and aVF. (b) An electrocardiogram after the patient became unresponsive shows narrow-complex tachycardia with premature ventricular contraction and ST-segment depression in I, II, and V5, V6. No P-waves were identified.

**Figure 2.**
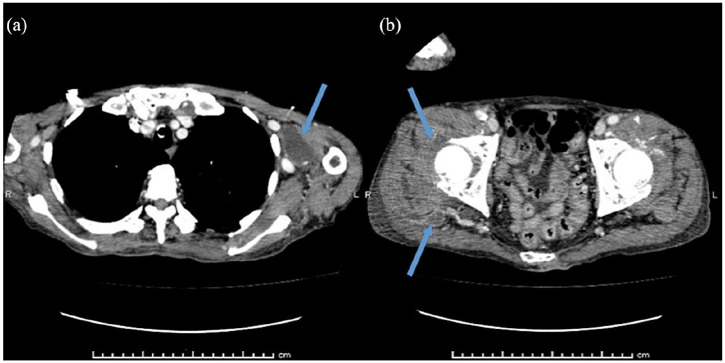
(a) Contrast-enhanced computed tomography (CT) shows a capsule-enclosed abscess in the left axilla (arrow). (b) Contrast-enhanced CT shows two capsule-enclosed abscesses in the right leg (arrows).

On arrival at the ER, vital signs were as follows: temperature, 38.3°C; heart rate, 90 beats/min with regular rhythm; respiratory rate, 36 breaths/min; blood pressure, 155/69 mmHg; and peripheral oxygen saturation, 97% in room air. Glasgow Coma Scale score was 13 (E4V3M6), indicating slight disturbance of consciousness due to pain.^9^ When he suddenly became unresponsive and his heart rate increased from 90 to 140 beats/min, a rapid response team was called immediately. He presented with hyperkalemia (potassium, 5.1 mEq/L (3.6–4.8 mEq/L)), increased C-reactive protein (34.94 mg/dL (0.00–0.14 mg/dL)), increased procalcitonin (17.36 ng/mL (0.00–0.05 ng/mL)), leukocytosis (13,920 cells/mL (3300–8600 cells/mL)) with neutrophilia (92.9%), abnormalities on electrocardiography (narrow-complex rhythm with ST-segment depression in leads II and V4–V6 (Figure 1(a)) changing to narrow-complex tachycardia with premature ventricular contraction and ST-segment depression in leads I, II, V5, and V6 (Figure 1(b)), and metabolic alkalosis with respiratory compensation (pH, 7.417; partial pressure of carbon dioxide (PCO_2_), 40.5 mmHg; partial pressure of oxygen (PO_2_), 80.0 mmHg; bicarbonate (HCO^3−^), 25.5 mmol/L; base excess, 0.9 mmol/L; and lactate, 1.93 mmol/L). Computed tomography (CT) showed multiple low-density areas, and contrast-enhanced CT showed disseminated abscess in the left axilla, right hip, and right leg (Figure 2(a) and (b)). He was transferred to the intensive care unit (ICU) on the day of admission and required intubation with mechanical ventilation.

**Figure 3.**
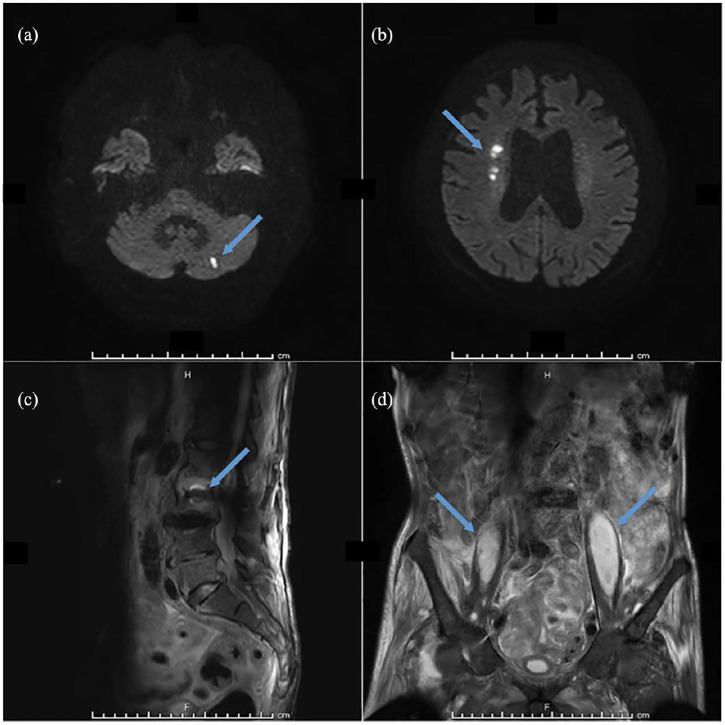
(a, b) Diffusion-weighted magnetic resonance imaging (MRI) reveals focally increased signal intensity in the left cerebellar hemisphere and right superior corona radiata (arrow), with septic emboli associated with methicillin-resistant *S. aureus* (MRSA) bacteremia difficult to distinguish from emboli due to atrial fibrillation. (c) Sagittal short tau inversion recovery (STIR)-weighted MRI shows focally increased signal intensity in the intervertebral disc between the L2 and L3 lumbar spines (arrow). (d) Coronal T2-weighted spectral attenuated inversion recovery (SPAIR) MRI shows bilateral iliopsoas muscle abscesses (arrows).

Antibiotics were started with meropenem, vancomycin, and gentamicin, as his condition was considered suggestive of IE. Three sets of blood cultures revealed methicillin-resistant *Staphylococcus aureus* (MRSA), so antibiotics were changed to ampicillin sulbactam, vancomycin, and gentamicin. Magnetic resonance imaging (MRI) of the brain showed multiple septic emboli (Figure 3(a) and (b)), and MRI of the lumbar spine showed infectious spondylodiscitis and iliopsoas abscess (Figure 3(c) and (d)). His condition gradually improved and he was successfully extubated and weaned off all inotropes, but frequently complained of intractable back pain with tachyarrhythmia. Acetaminophen administered either by drip infusion or orally failed to effectively mitigate the back pain. Tachyarrhythmia was also persistent whenever LBP was strong (numerical rating scale was 10) and could not be managed with either cardioversion or amiodarone. Fentanyl (0.4 μg/kg/h) was initiated on day 19, with the intractable pain and tachyarrhythmia both resolving shortly thereafter. He was transferred to a general ward, but experienced repeated episodes of fever that proved unresponsive to multiple regimens of antibiotics. The patient died on day 50 of hospitalization. Sputum culture revealed extended-spectrum beta-lactamase (ESBL)-producing *Escherichia coli*.

## Discussion

We encountered a case in which pain management with fentanyl improved intractable tachyarrhythmia resulting from infectious spondylodiscitis with IE. Several antiarrhythmic medications and cardioversion offered limited efficacy for short periods, and pain management offered the most effective treatment for intractable tachyarrhythmia in this case.

Infectious spondylodiscitis is uncommon, but sometimes causes serious LBP. Our patient suffered from persistent intractable LBP and an orthopedic surgeon had examined him several times, but the patient had not been scrutinized because of the absence of vital sign abnormalities for a month, and he also had complained of back pain due to lumbar spinal canal stenosis for long time. The fact that LBP represented serious infectious spondylodiscitis should have been identified and noted. The disseminated abscess with infectious spondylodiscitis that seemed to have caused IE or vice versa was confirmed using Duke criteria according to the presence of two major criteria and three minor criteria,^10^ although multiple performances of transthoracic echocardiography (TTE) failed to identify vegetation (the patient refused transesophageal echocardiography). IE was managed well by anti-MRSA drugs in rotational use, but these antibiotics led to microbial substitution with ESBL and the patient finally died of *E. coli* pneumonia.

Although evidence for the utility of opioid analgesics in acute LBP is limited,^11^ one study showed that fentanyl administered at 4 μg/kg minimized the hemodynamic changes and prevented tachycardia under processing of tracheal intubation.^12^ In our case, however, fentanyl management at 0.4 μg/kg/h relieved him from LBP. Possible reasons for this discrepancy were as follows: his thin, small body (height, 166.5 cm; weight, 47.7 kg; body mass index, 17.2 kg/m^2^); renal impairment; and previous insufficient analgesic usage with acetaminophen and non-steroidal anti-inflammatory drugs.

## Conclusion

Infectious spondylodiscitis should be taken into account when a patient presents with several high-risk factors including HD, older age, presence of an implanted device, and persistent pain with fever. Pain management may help control tachycardia when antiarrhythmic medications prove ineffective.
